# Newly Diagnosed Atrial Fibrillation Is an Independent Factor for Future Major Adverse Cardiovascular Events

**DOI:** 10.1371/journal.pone.0123211

**Published:** 2015-04-15

**Authors:** Chen-Yu Li, Chia-Pin Lin, Yu-Sheng Lin, Lung-Sheng Wu, Chee-Jen Chang, Pao-Hsien Chu

**Affiliations:** 1 Department of Cardiology, Chang Gung Memorial Hospital, Chang Gung University College of Medicine, Taipei, Taiwan; 2 Healthcare Center, Chang Gung Memorial Hospital, Chang Gung University College of Medicine, Taipei, Taiwan; 3 Clinical Informatics and Medical Statistics Research Center, College of Medicine, Chang Gung University, Taipei, Taiwan; 4 Heart Failure Center, Chang Gung Memorial Hospital, Chang Gung University College of Medicine, Taipei, Taiwan; University of Perugia, ITALY

## Abstract

**Objectives:**

This study aims to investigate the impact of newly diagnosed atrial fibrillation (AF) on future major adverse cardiac events (MACE). AF is the most common form of cardiac arrhythmia and is associated with several other cardiovascular (CV) events. Little is known about whether newly diagnosed AF is an independent factor for future MACE, especially in patients without such a history.

**Methods and Results:**

We evaluated data from the National Health Insurance Research Database, which represented a retrospective cohort of 713,288 adults in Taiwan from 2006 to 2010. Individuals with previous MACE were excluded. Newly diagnosed AF patients were identified by assigning International Classification of Diseases codes. Propensity score matching adjusted for gender, age, hypertension, diabetes mellitus and dyslipidemia. Cox proportional hazard models estimated future MACE ratios. We compared a total of 3,737 patients with newly diagnosed AF and 704,225 patients without. After matching, there was no difference in baseline demographic characteristics in patients across newly diagnosed AF and non-AF groups. The result showed that newly diagnosed AF in multivariate analysis were associated with increased incidents of MACE (hazard ratio: 3.11-3.51 in different models) and mortality. Newly diagnosed AF without other CV risk factors had 8.45 times the risk of developing future MACE than healthy adults. The more associated CV risk factors in addition to AF, the increased rate of future CV events.

**Conclusions:**

Newly diagnosed AF is an independent factor that leads to future CV events after gender, age, hypertension, diabetes mellitus and dyslipidemia matching. AF is associated with a higher mortality rate.

## Introduction

Atrial fibrillation (AF) is the most common cardiac arrhythmia and affects more than 1.2% of the general population [1]. AF is a well-documented independent risk factor for stroke [2–5], heart failure (HF) [2, 6], and premature death [2, 6–12]. Meanwhile, AF in patients with cardiac comorbidities, such as HF with or without left ventricular dysfunction [10, 13] and myocardial infarction [8], are also associated with an increased risk of cardiovascular (CV) events and mortality. In previous studies, newly diagnosed AF was specifically analyzed and also disclosed a higher risk of mortality [9, 11]. However, little is known about the influence of newly diagnosed AF on major adverse cardiovascular events (MACE), such as myocardial infarction (MI), percutaneous coronary intervention (PCI), coronary artery bypass grafting (CABG), HF, stroke, malignant dysrhythmia, thrombolysis and cardiogenic shock, especially in patients without pre-existing events. Furthermore, all of the population-based studies, to our knowledge, were conducted in Western countries and most of the patients were of Caucasian decent. Previously, we have studied the effect of MACE in different populations [14–16]. In this study, we analyzed a large-scale, population-based data in an Asian population from National Health Insurance (NHI) claims records in Taiwan to evaluate the impact of newly diagnosed AF on future CV events in adults without pre-existing MACE.

## Methods

Informed consent was waived as the database analysis used de-identified secondary data, and the study was approved by the Institutional Review Board of Chang Gung Memorial Hospital (#98-4060B). All the patient records/information was anonymized and de-identified prior to analysis.

### Data Source

The NHI in Taiwan started in March 1995 and provides effective insurance coverage to the entire population. Approximately 96% of the Taiwanese population has registered for the NHI program [17]. Since 1996, the National Health Insurance Research Database (NHIRD) has covered 97% of hospitals and clinics throughout the country [18].

**Fig 1:**
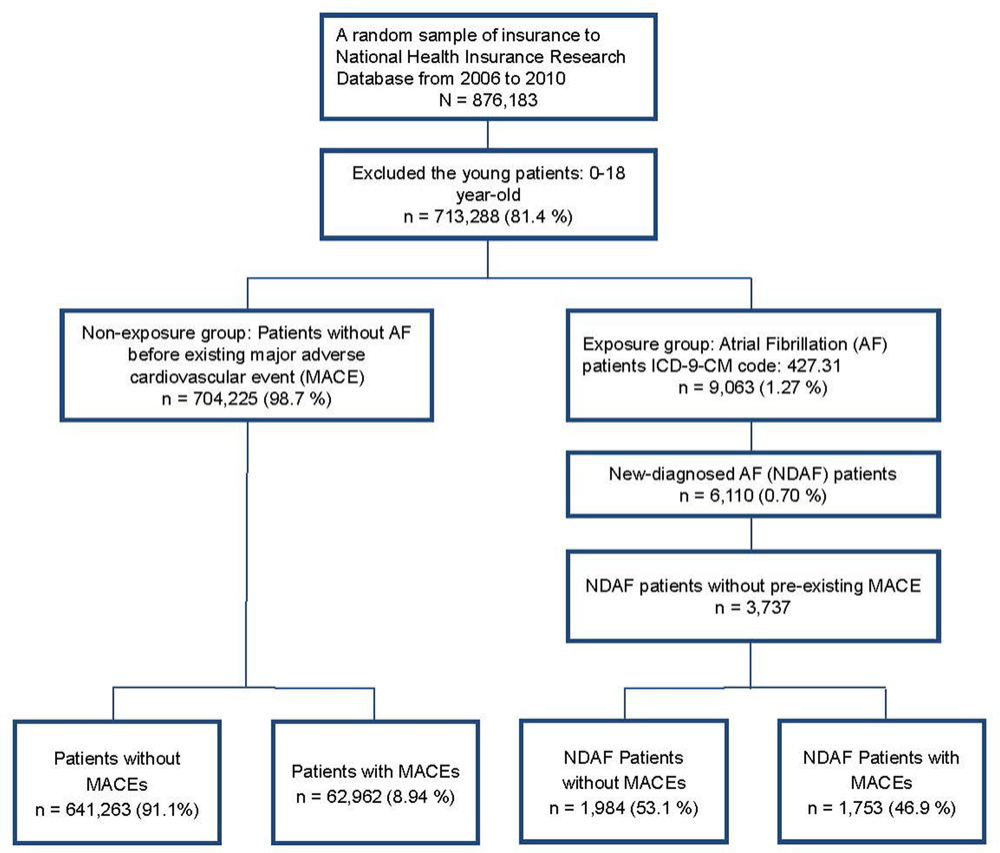
Flowchart of the relationship between newly diagnosed AF and MACE. AF = atrial fibrillation; MACE = major adverse cardiovascular events.

Data on the prevalence and incidence of AF in Taiwanese adults were obtained from the Department of Household Registration Affairs between 2006 and 2010. Randomized NHIRD data from the same period were used for the current study. After excluding individuals younger than 18 years of age, a total of 713,288 participants were analyzed (Fig 1). AF was diagnosed by International Classification of Diseases-9-Clinical Modification (ICD-9-CM) code: 427.31, where gender, age, and medical treatment information were also considered. Patients of newly diagnosed AF without pre-existing MACE were acquired by ICD-9-CM coding during this period (n = 3,737).

In order to effectively investigate the relationship between newly diagnosed AF and future MACE, pre-existing events were also excluded, including myocardial infarction (MI, ICD-9-CM code: 410–410.9), PCI (operation code: 36.0–36.03, 36.05–36.09), CABG (operation code: 36.1–36.99, V45.81), HF (ICD-9-CM code: 428.0–428.10), stroke (ICD-9-CM code: 430–437), malignant dysrhythmia (IDC-9-CM code: 426.0, 426.12–426.13, 426.51, 426.52, 426.54, 427.1, 427.4, 427.41, 427.42, 427.5), thrombolysis (operation code: 36.0–36.99), cardiogenic shock (ICD-9-CM code: 785.51), pulmonary embolism (ICD-9-CM code: 415.1, 415.11, 415.19, 673), and deep vein thrombosis (ICD-9-CM code: 453.0, 453.2, 453.3, 453.8).

### Case matching

In Framingham Heart Study, only age, gender, total cholesterol, high density lipoprotein cholesterol, smoking and systolic blood pressure were used to predict future cardiac events [19]. So we used propensity score matching of SAS macro at a ratio of 4:1 to adjust the influences of other associated CV risk factors, such as hypertension (ICD-9-CM code: 401–405, 437.2, and 362.11), diabetes mellitus (DM, ICD-9-CM code: 250, 357.2, 362.01, 362.02, and 366.41), dyslipidemia (ICD-9-CM code: 272), gender, and age (older than 65 years).

### Statistical analysis

Chi-square tests were used to compare categorical variables and Student’s t-test was employed for continuous variables. The Kaplan-Meier method was also used to estimate overall survival, and the log-rank test was used to test the difference between groups. The Cox model was used to estimate covariate values, such as gender, classified age, elderly (≥65 years), AF, hypertension, DM, and dyslipidemia. Finally, both Score and Wald test were considered to verify that these Cox models with parameters can appropriately be estimated from the sample in NHIRD. The criterions for model selection, such as Akaike information criterion (AIC) and Bayesian information criterion (BIC), were adopted for selecting the outcome models from a set of candidate models as well. Data were calculated as means, standard deviations, percentages and confidence intervals. All analyses were conducted by using SAS statistical software, Version 9.3 (SAS Institute Inc., Cary, North Carolina). A p-value < 0.05 was considered statistically significant.

## Results

**Fig 2:**
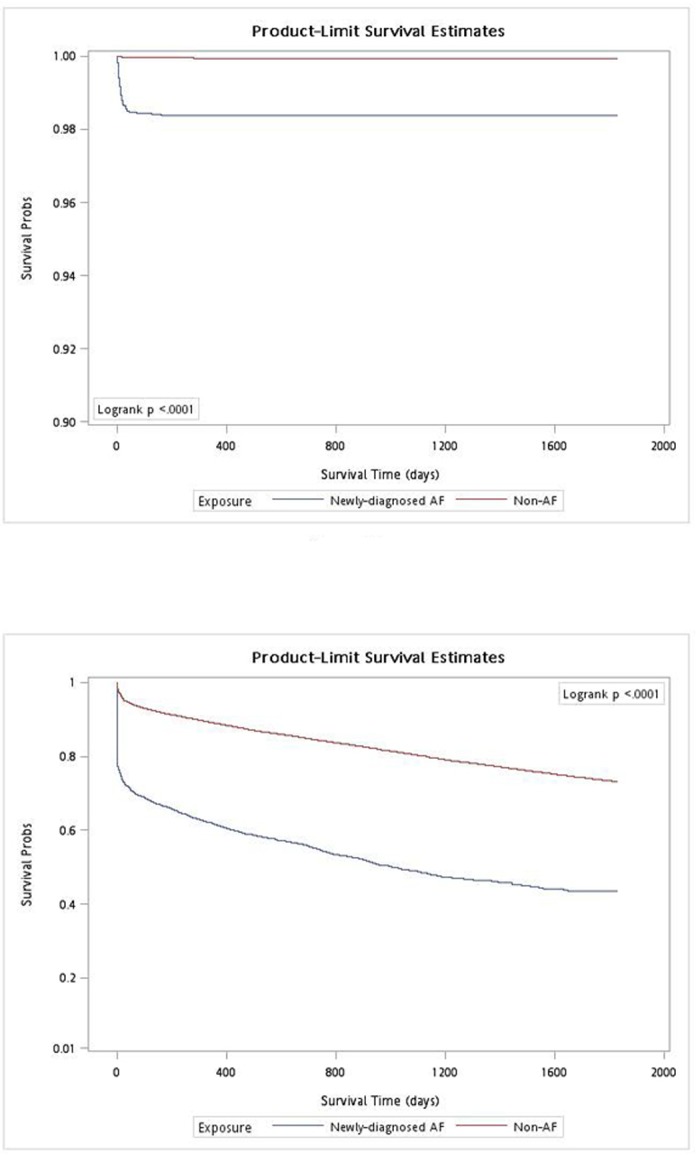
Mortality and MACE between AF and non-AF. The Kaplan-Meier estimated cumulative all-cause death in non-atrial fibrillation (AF, red) and newly diagnosed AF (blue). A. Mortality; B. Major adverse cardiovascular events (MACE).

The prevalence and incidence of AF in Taiwanese adults increases with age in both men and women (Table 1). After exclusion, a total of 704,225 participants were identified who did not have previous CV events and AF, compared to a total of 3,737 participants who developed newly diagnosed AF without pre-existing CV events (Table 2). Before matching, the differences of baseline characteristics—gender, age, hypertension, DM and dyslipidemia—were significantly more in the AF group (*P*<0.0001) than the non-AF group. The incidences of future MACE and mortality were also predominant in the AF group (*P*<0.0001). After matching at a ratio of 4:1, the differences of the baseline characteristics were identical in both groups (*P* = 1), while future MACE and mortality remained more frequent in the AF group (*P* = 0.0113 to *P*<0.0001), and most of the deaths were non-stroke related in both groups. Among these MACE, HF was the most frequent developed event in AF group (27.1%) followed by stroke (21.9%). However, in non-AF group, stroke was the most frequent developed event (19.5%) instead of HF (7.81%). Moreover, both MACE and mortality were significantly different between AF and non-AF groups over time (Fig 2, both Log-rank *P*<0.0001) when analyzed by using Kaplan-Meier method.

**Table 1:** The prevalence and incidence of atrial fibrillation (AF) between 2006 and 2010 in Taiwan.

	Population		Prevalence of AF		Incidence of AF	
Age	Female	Male	Female	Male	Female	Male
18–55	6,701,788	6,776,865	9,363	17,115	5,200	9,237
(%)			(0.14)	(0.25)	(0.08)	(0.14)
55–65	1,325,396	1,274,414	12,589	19,412	5,709	8,262
(%)			(0.95)	(1.52)	(0.43)	(0.65)
65–75	735,413	650,323	25,441	29,831	8,840	10,085
(%)			(3.46)	(4.59)	(1.20)	(1.55)
75–85	438,191	423,123	33,535	40,625	8,720	11,101
(%)			(7.65)	(9.27)	(1.99)	(2.62)
85+	125,778	115,065	16,145	13,042	3,557	3,181
(%)			(12.8)	(11.3)	(2.83)	(2.76)
Total	9,326,566	9,239,790	97,073	120,025	32,026	41,866
(%)			(0.10)	(0.13)	(0.34)	(0.45)

Note: Source from Department of Household Registration Affairs, Ministry of the Interior, ROC. 2010. The official website is http://sowf.moi.gov.tw/stat/year/list.htm.

**Table 2:** Matching by Propensity Score method in participants with and without newly diagnosed atrial fibrillation (AF).

	Before matching					After matching				
Data Source	Without AF		With AF		*P* Value	Without AF		With AF		*P* Value
	(n = 704,225)		(n = 3,737)			(n = 14,948)		(n = 3,737)		
	n	(%)	n	(%)		n	(%)	n	(%)	
Gender					<0.0001					1.0000
Female	354,651	(50.4)	1,659	(44.4)		6,636	(44.4)	1,659	(44.4)	
Male	349,574	(49.6)	2,078	(55.6)		8,312	(55.6)	2,078	(55.6)	
Elder	89,044	(12.6)	2,345	(62.8)	<0.0001	9,380	(62.8)	2,345	(62.8)	1.0000
HTN	176,682	(25.1)	2,316	(62.0)	<0.0001	9,264	(62.0)	2,316	(62.0)	1.0000
DM	91,949	(13.1)	945	(25.3)	<0.0001	3,780	(25.3)	945	(25.3)	1.0000
Dyslipidemia	138,514	(19.7)	899	(24.1)	<0.0001	3,596	(24.1)	899	(24.1)	1.0000
MACE	62,962	(8.94)	1,753	(46.9)	<0.0001	4,006	(26.8)	1,753	(46.9)	<0.0001
MI	7,270	(1.03)	219	(5.86)	<0.0001	502	(3.36)	219	(5.86)	<0.0001
PCI	4,378	(0.62)	149	(3.99)	<0.0001	252	(1.69)	149	(3.99)	<0.0001
CABG	985	(0.14)	34	(0.91)	<0.0001	64	(0.43)	34	(0.91)	0.0003
HF	14,996	(2.13)	1,013	(27.1)	<0.0001	1,167	(7.81)	1,013	(27.1)	<0.0001
Stroke	46,119	(6.55)	818	(21.9)	<0.0001	2,911	(19.5)	818	(21.9)	0.0010
Malignant arrhythmia	2,804	(0.40)	132	(3.53)	<0.0001	186	(1.24)	132	(3.53)	<0.0001
Thrombolysis	4,378	(0.62)	149	(3.99)	<0.0001	252	(1.69)	149	(3.99)	<0.0001
Cardiogenic shock	692	(0.10)	52	(1.39)	<0.0001	54	(0.36)	52	(1.39)	<0.0001
Pulmonary embolism	625	(0.09)	21	(0.56)	<0.0001	37	(0.25)	21	(0.56)	0.0020
Deep vein thrombosis	1,947	(0.28)	38	(1.02)	<0.0001	94	(0.63)	38	(1.02)	0.0113
Mortality	151	(0.02)	60	(1.61)	<0.0001	8	(0.15)	60	(1.61)	<0.0001
Stroke death	18	(11.9)	10	(16.7)	<0.0001	0	(0.0)	10	(16.7)	<0.0001
Non-stroke death	133	(88.1)	50	(83.3)	<0.0001	8	(100.0)	50	(83.3)	<0.0001

Elder = the people are over 65 years old; AF = atrial fibrillation; CABG = coronary artery bypass grafting; DM = diabetes mellitus; HF = heart failure; HTN = hypertension; MACE = major adverse cardiovascular events; MI = myocardial infarction; PCI = percutaneous coronary intervention.

Univariate Cox regression analysis revealed that female, elderly, AF, hypertension, DM and dyslipidemia patient tended to develop more MACE statistically (Table 3). In order to describe the effects of age level in MACE, it was organized into category (18–55, 55–65, 65–75, 75–85, and 85+ years) and binary (over 65 years or not) data to depict in multivariate model 1 and 2, respectively. In multivariate models with satisfying the criteria of model selection, similar results were obtained for elderly (hazard ratio, HR 2.98–9.77, *P*<0.0001 in Model 1 and HR 3.4, *P*<0.0001 in Model 2), AF (HR 3.11–3.51, *P*<0.0001), hypertension (HR 1.85–2.03, *P*<0.0001) and DM patients (HR 1.32–1.33, *P*<0.0001) (Table 4). However, males developed more future MACE (HR 1.11–1.12, *P*<0.0001) in multivariate analysis, and there was statistically insignificant in dyslipidemia in Model 2 (HR 1.04, *P* = 0.1661). The multivariate Cox modeling and model selection were showed in Table 5, which showed both models were appropriately fitted for the sample in statistical significance. We further demonstrated the average time between the incident of AF and MACE (Table 6), which showed the earliest developed MACE after newly diagnosed AF were MI and HF (0.49 year, 95% CI 0.36 to 0.62 and 0.44 to 0.54, respectively). The latest developed event was malignant arrhythmia (1.09 year, 95% CI 0.89 to 1.29).

**Table 3:** Univariate Cox regression analysis for factors and 2x2 tables associated with MACE.

Variables	MACE				Univariate analysis		
	No	(%)	Yes	(%)	HR	95%CI	*P value*
Gender							
Female	5,671	(68.4)	2,624	(31.6)	1.0		
Male	7,255	(69.8)	3,135	(30.2)	0.95	0.90–1.00	0.0424
Age							
18–55	4,752	(92.7)	375	(7.31)	1.0		
55–65	1,323	(72.2)	510	(27.8)	4.65	4.07–5.31	<0.0001
65–75	4,249	(68.1)	1,992	(31.9)	5.01	4.49–5.59	<0.0001
75–85	2,175	(50.4)	2,143	(49.6)	9.00	8.06–10.0	<0.0001
85-	427	(36.6)	739	(63.4)	13.1	11.6–14.8	<0.0001
Elder							
No	6,075	(87.3)	885	(12.7)	1.0		
Yes	6,851	(58.4)	4,874	(41.6)	3.84	3.58–4.13	<0.0001
AF							
No	10,942	(73.2)	4,006	(26.8)	1.0		
Yes	1,984	(53.1)	1,753	(46.9)	3.40	3.20–3.60	<0.0001
HTN							
No	5,875	(82.7)	1,230	(17.3)	1.0		
Yes	7,051	(60.9)	4,529	(39.1)	2.58	2.42–2.74	<0.0001
DM							
No	10,162	(72.8)	3,798	(27.2)	1.0		
Yes	2,764	(58.5)	1,961	(41.5)	1.67	1.59–1.77	<0.0001
Dyslipidemia							
No	9,992	(70.4)	4,198	(29.6)	1.0		
Yes	2,934	(65.3)	1,561	(34.7)	1.21	1.14–1.28	<0.0001

Elder = the people are over 65 years old; AF = atrial fibrillation; DM = diabetes mellitus; HR = hazard ratio; HTN = hypertension; MACE = major adverse cardiovascular events.

**Table 4:** Multivariate Cox models for factors associated with MACE.

Variables	Model 1			Model 2		
	HR	95%CI	*P* value	HR	95%CI	*P* value
Gender						
Female	1.0			1.0		
Male	1.12	1.06–1.18	<0.0001	1.11	1.06–1.17	<0.0001
Age (year)						
18–55	1.0			1.0		
55–65	2.98	2.60–3.41	<0.0001			
65–75	4.21	3.77–4.71	<0.0001	3.40	3.16–3.66	<0.0001
75–85	6.76	6.05–7.56	<0.0001			
85+	9.77	8.61–11.1	<0.0001			
AF						
No	1.0			1.0		
Yes	3.11	2.93–3.30	<0.0001	3.51	3.31–3.72	<0.0001
HTN						
No	1.0			1.0		
Yes	1.85	1.73–1.97	<0.0001	2.03	1.90–2.17	<0.0001
DM						
No	1.0			1.0		
Yes	1.32	1.25–1.40	<0.0001	1.33	1.25–1.41	<0.0001
Dyslipidemia						
No	1.0			1.0		
Yes	1.13	1.06–1.20	0.0001	1.04	0.98–1.11	0.1661

AF = atrial fibrillation; DM = diabetes mellitus; HR = hazard ratio; HTN = hypertension; MACE = major adverse cardiovascular events.

**Table 5:** Multivariate Cox modeling and model selection.

	Model 1		Model 2	
Modeling				
	Coefficient	*P* value	Coefficient	*P* value
Score	4,925	<0.0001	4,196	<0.0001
Wald	4,020	<0.0001	3,734	<0.0001
-2 log L.	106,147		106,813	
Model selection				
AIC	106,165		106,825	
BIC	106,225		106,865	

-2 log L. = -2 log Likelihood; AIC = Akaike information criterion; BIC = Bayesian information criterion; Score and Wald are testing global hull hypothesis by Chi-square test with 9 and 6 degree of freedom in model 1 and 2, respectively.

**Table 6:** The average time between the incident of AF and MACE.

	AF (n = 3,737)		
MACE (n = 1,753)	n	years	95%CI
MI	219	0.49	0.36–0.62
PCI	149	0.61	0.47–0.75
CABG	34	1.06	0.55–1.57
HF	1,013	0.49	0.44–0.54
Stroke	818	0.58	0.52–0.64
Malignant arrhythmia	132	1.09	0.89–1.29
Thrombolysis	149	0.60	0.47–0.73
Cardiogenic shock	52	0.59	0.36–0.82
Pulmonary embolism	21	0.91	0.34–1.48
Deep vein thrombosis	38	0.57	0.35–0.79

AF = atrial fibrillation; MACE = major adverse cardiovascular events; MI = myocardial infarction; PCI = percutaneous coronary intervention; CABG = coronary artery bypass grafting; HF = heart failure; CI = confidence interval.

**Fig 3:**
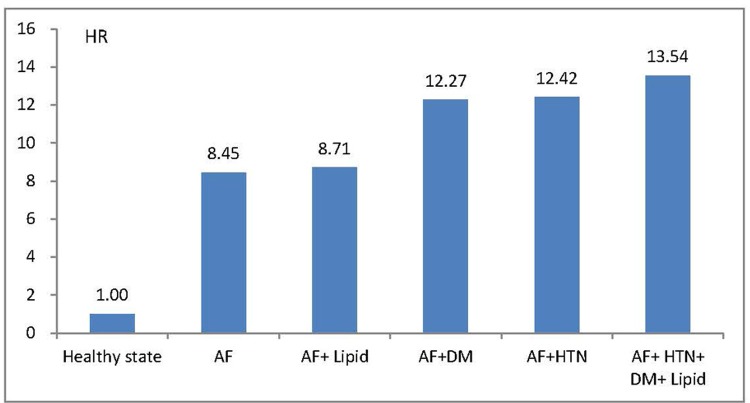
HR for future MACE. The hazard ratios (HR) of atrial fibrillation (AF) and other cardiovascular risk factors compared to healthy participants in developing future major adverse cardiovascular events (MACE). Lipid = dyslipidemia; DM = diabetes mellitus; HTN = hypertension.

Moreover, compared to healthy participants without any CV risk factors, newly diagnosed AF was associated with 8.45 times the risk of developing future MACE (95% CI 7.37 to 9.68, *P*<0.0001) (Fig 3). In addition to AF, the rate of future MACE increased when patients had more associated CV comorbidities (HR 8.71, 95% CI 6.55 to 11.58, *P*<0.0001 in AF and dyslipidemia; HR 12.27, 95% CI 9.51 to 15.82, *P*<0.0001 in AF and DM; HR 12.42, 95% CI 10.94 to 14.09, *P*<0.0001 in AF and hypertension; and HR 13.54, 95% CI 11.29 to 16.22, *P*<0.0001 in AF with hypertension, DM and dyslipidemia).

## Discussion

In previous studies, the influence of AF on future MACE or premature death did not exclude co-existing CV diseases. The present work is the first large-scale study to show that newly diagnosed AF patients without prior CV diseases associate with a higher risk of MACE, compared to patients without AF. After age, gender, hypertension, DM and dyslipidemia matching, newly diagnosed AF remains an independent risk factor for future MACE. In addition, AF is also a strong predictor of mortality over time.

In a subgroup analysis of the Candesartan in heart failure—assessment of reduction in mortality and morbidity (CHARM) program, AF in patients with HF experienced a higher risk of worsening HF and stroke regardless of baseline ejection fraction [10]. In the same study, newly onset of AF patients with HF was also associated with worse CV outcomes. Similar results were also demonstrated in a recently published study [13]. The relationship between long-term outcome and AF in patients after acute MI complicated by HF was also examined in a subgroup analysis of Valsartan in acute myocardial infarction trial (VALIANT) [8]. Both previously known and newly diagnosed AF were associated with more CV events than those without AF. However, in another population-based study in Scotland, AF was associated with more stroke and HF events but not acute MI [6]. In our study, we excluded pre-existing CV diseases in the new onset of AF, which is different from previous studies. Our study demonstrated a higher risk not only for HF and stroke but also for MI, PCI, CABG, malignant dysrhythmia, thrombolysis and cardiogenic shock.

As expected, age, hypertension and DM were also associated with higher risk of MACE in our multivariate analysis. Similar results have been well established in previous studies [6, 11, 20, 21], and treating such factors can significantly reduce future events [22]. However, the impact of gender on MACE is uncertain. For stable angina but normal coronary arteries, the MACE rate was equal among men and women [23]. In two population-based studies, cardiovascular events and mortality were more frequently developed in women in one [6] but not in the other [11]. In current study, we demonstrated that men associated with a higher MACE rate than women. We suspect the uncertainty may come from different selected cohorts and population.

AF is an independent factor for mortality and future thromboembolic events [2–4]. On the other hand, previous study showed the majority of deaths in AF patients were not related to stroke [24]. In our study, we also demonstrated most of the mortality were non-stroke related in both AF and non-AF groups. Mortality was higher in permanent and first detected AF compared to paroxysmal and persistent AF [25]. In the past, AF management concepts have changed, such as the increased use of antiarrhythmic drugs, control underlying comorbidities and increased aggressiveness in anticoagulation and AF ablation. Mortality trends have improved over time in some studies [26–28] but not in others [9]. A community-based study concluded that newly diagnosed AF was associated with high mortality risk, especially within the first four months, but the mortality trend has not significantly changed over the past 21 years [9]. The authors presumed that AF per se may act as a “risk marker” rather than a “risk factor”. The worse outcome resulted from underlying diseases or pathologic changes, and the treatment toward AF itself may have not influenced the mortality trend. Similar points of view were also proposed by another study [11] in which patients who had new-onset AF died earlier than the general population but mainly due to non-CV causes. The mortality trend was constant for both in patients with or without previous heart diseases. The authors concluded that AF itself may be only a minor component of excess mortality. In our study, newly diagnosed AF without pre-existing CV diseases was associated with higher mortality rate than in patients without AF. We speculate that the cause of premature death is multifactorial, and new-onset AF itself may be not the direct cause but represent a higher severity of underlying diseases, which results in higher mortality. Treatment of the primary medical illnesses that lead to new-onset AF may be the first priority to reduce the risk of premature death.

Potential limitations of this study should be noted. First, the diagnosis of AF was made by the ICD-9 code from NHIRD but not directly by documented electrocardiography. Although we compared the results with the patients without such coding, there may exist some misclassified cases in either group. In addition, the sustaining pattern of newly developed AF was not recorded in our database, and therefore we could not evaluate the different impacts among permanent, persistent and paroxysmal AF patients. Second, several unmeasured confounders, such as body mass index, smoking, blood pressure level or low density lipoprotein level, which are associated with MACE and CV mortality, were not included in our database. Third, the incidence of MACE was also collected from ICD-9 and operation codes, where the details of such events were not available, such as ST-segment elevation MI or non-ST-segment elevation MI, systolic or diastolic HF, and the numbers of coronary arteries that intervened. Although the NHI system includes almost all of the hospitals and clinics in Taiwan, it is possible that some cases of MACE or mortality did not seek any medical attention. Finally, the study was a population-based cohort study in Taiwan in which the results may not be applicable to other ethnic and racial groups.

In conclusion, newly diagnosed AF without pre-existing MACE is an independent factor for future CV events even after matching with other associated CV risk factors. Compared to patients without AF, newly diagnosed AF is also associated with a higher mortality risk. In addition to AF, more associated CV comorbidities increase the rate of future MACE. Further interventional studies to treat newly onset AF itself are needed to determine whether AF is a primary cause or a risk marker of premature death.
